# Microbiological control project of minced meat of wild boar (*Sus scrofa* L.) in approved establishments in Sweden

**DOI:** 10.1186/s13028-025-00840-7

**Published:** 2025-12-04

**Authors:** Arja Helena Kautto, Åsa Rosengren, Catarina Flink, Cathrine Domeij

**Affiliations:** 1 Department of Animal Biosciences, Swedish University of Agricultural Sciences, Ulls Väg 26, Uppsala, Sweden; 2 Control Support Unit, Department of Food Control, Swedish Food Agency, Dag Hammarskjöldsvägen 56 A, 75126 Uppsala, Sweden; 3 Division for Advice and Regulation, Unit for Food Hygiene, Swedish Food Agency, Dag Hammarskjöldsvägen 56 A, 75126 Uppsala, Sweden

**Keywords:** Aerobic colony count, *Escherichia coli*, Game handling, Microbiological criteria, Risk management, *Salmonella* spp.

## Abstract

The aims of this study were to enhance knowledge of microbiological status of minced meat of Swedish wild boar and to evaluate its compliance with the Commission Regulation (EC) No 2073/2005 on microbiological criteria for foodstuffs. The sampling was performed by the official control staff at Swedish Food Agency during 2024. While wild boar has the capacity to harbor a diverse range of zoonotic agents there is a lack of microbiological data on the products derived from wild boar carcasses. More information concerning the microbiological status of minced meat of wild boar is needed. These results could contribute to the development of risk-based management strategies and the verification of control measures through the safe game meat chain. One sample from each of selected 33 approved establishments producing minced meat of wild boar was sent to a commercial laboratory accredited for the analyses. Process hygiene criteria Aerobic Colony Count (ACC) and *Escherichia coli* were analysed as well as food safety criteria *Salmonella* spp. and results were assessed according to the microbiological criteria for minced meat. Results show no presence of *Salmonella* spp. in any of the samples. The 165 units, clustered in 33 plants, had a median for ACC 5.6 log_10_ colony forming units, cfu/g and *E. coli* 1.8 log_10_ cfu/g. However, 25 of 33 samples (proportion 0.758, 95% Confidence interval, CI: 0.577;0.889) were unsatisfactory. In total, about one fourth of samples, (proportion 0.242, 95% C.I: 0.111;0.423) were satisfactory or acceptable. The evidence for a temporal trend from September to December was insufficient. The results show non-compliance with the process hygiene criteria for minced meat of wild boar. Hunters must be informed about the key importance of proficient shooting skills and adequate evisceration procedures. Food business operators must focus on control of incoming carcasses and hygienic handling in every stage of the production. In case of unsatisfactory results according to in-house sampling, effective corrective action should be implemented by food business operator and verified by official control. Our results show that it is possible to produce minced meat of wild boar of good bacteriological quality.

## Findings

Food business operators have the main responsibility for the food safety released on the market [1] including microbiological criteria. The official controls must verify that food business operators’ performances are compliant with the actual regulations [2, 3]. The aims of the project were to enhance knowledge of microbiological status of minced meat of Swedish wild boar, evaluate its compliance with the the Commission Regulation (EC) No 2073/2005 on microbiological criteria for foodstuffs and to train the control personnel to use sampling as a control method according to the regulation [4].

In the governmental document of Sweden’s National Food Strategy [5] wild game is pointed out as an important source of food. Government owned Wild Boar Package [6] with economic support to enhance wild boar hunting and consumption is encouraging many consumers to buy and consume more wild boar meat. However, wild boars are reported as carriers of several zoonotic agents such as *Trichinella* spp., *Salmonella* spp., *Yersinia* spp. *Toxoplasma gondii*, hepatitis E-virus and occasionally shiga toxin-producing *E. coli* (STEC) [7–10]. Wild boar meat can pose a number of microbiological risks if not handled and processed in a secure manner. Several factors such as shot placement, evisceration skills [11] and storage temperature affect the hygienic level of the meat. Hence, the level of contamination varies a lot between carcasses [12].

Important parameters to analyse for meat safety and quality are Salmonella, Aerobic Colony Count (ACC) and *Escherichia coli.* The Commission Regulation (EC) No 2073/2005 on microbiological criteria for foodstuffs defines process hygiene and food safety criteria [4]. Process hygiene criteria set acceptable contamination levels during production. If exceeded, corrective actions to maintain or restore process hygiene are needed. These criteria apply at the end of production before products reach the market. Food safety criteria define when food that already is on the market is safe. If a criterion is exceeded, the food is considered unsafe and must be withdrawn or if possible, reprocessed to eliminate hazards. For minced meat, including minced meat of wild boar, ACC and *E. coli* are prescribed as process hygiene criteria and *Salmonella* spp. is defined as a food safety criterion [4].

ACC provides indication of the food’s general condition, but it reveals neither spoilage nor pathogenic microorganisms. Therefore, high ACC in foods does not necessarily pose a health risk [13]. However, it may indicate insufficient process hygiene due to e.g. early spoilage, poor-quality raw materials, unhygienic handling conditions, insufficient cooling, or inappropriate time/temperature storage [14]. *Escherichia coli* is a very common bacteria in the intestinal tract of warm-blooded animals, including humans. Thus, it serves as an indicator of faecal contamination in meat and other foods, including drinking water [13]. Contamination of *E. coli* on wild boar meat can occur during hunting, evisceration and slaughter procedures. Insufficient hand hygiene among personnel and inadequately sanitized surfaces in food-processing environments increase risk for contamination [15, 16]. Detection of generic *E. coli* does not inherently pose a direct public health risk. However, certain strains, particularly shiga toxin-producing *E. coli* (STEC) can cause severe human illness [17]. STEC in minced meat is not included in the Regulation (EC) No 2073/2005 criteria.

*Salmonella* spp. originate in the intestines and can be present in both animal- and plant-based foods. In animal derived products, *Salmonella* spp. usually stems from infected animals. In food facilities, environmental strains may contaminate food products [18]. Presence of *Salmonella* spp. in food may pose a health risk [4]. Certain *Salmonella* serotypes are specifically adapted to animal species. For instance, *Salmonella choleraesuis* is particularly adapted to swine including both domestic pigs and wild boar [19].

Sampling and analysis were done September-December 2024 at 33 of 96 (35%) Swedish production plants approved for mincing wild boar meat. These 33 plants were the ones in production during the sampling period. No other criteria were used for choosing the plants in the study. At each plant, one frozen sample (− 18 °C) á 500 gr consisting of five 100-gram units was taken. Samples of frozen packed minced meat were taken directly or sent by express mail (maximum 8 h, packed in cooler bag) to an official laboratory accredited for the analyses (SWEDAC, SS-EN ISO/IEC 17025:2018, accreditation number 1006) [20]. After arrival at the laboratory, samples were thawed for eight to twelve hours at maximum 4 °C and thereafter directly analysed. In total, 165 sample units á 100 g were analysed [4]. ACC and *E. coli* were analysed according to current versions of methods published by the Nordic committee on food analyses (NMKL) [21]. Five samples á 25 g were analysed for *Salmonella* spp. according to the VIDAS^®^ Salmonella (SLM), 48 h protocol (Afnor Certificate number: BIO 12/10−09/02) (BioMèrieux). Assessment of the result was done according to the microbiological criteria for minced meat [4].

The results indicate that *Salmonella spp*. was not present in the examined samples. Consequently, the minced meat met the legal Food safety criteria for *Salmonella spp.* according to the Chap. 1.1.6 [4]. However, 25 of 33 samples (0.758, 95% Confidence interval, CI: 0.577;0.889) were unsatisfactory, mainly due to high levels of *E. coli* assessed according to Chap. 2.1.6. for Process hygiene criteria [4]. Freezing causes cellular damage and Gram-negative bacteria are particulary susceptible. Therefore, bacterial counts of *E. coli* and ACC are most likely underestimated [22].

The number of samples assessed as satisfactory/acceptable versus unsatisfactory for both ACC and *E. coli* are presented separate and together in Table 1.

**Table 1:** Number of samples assessed* for *Escherichia coli* and aerobic colony count (ACC)

Production volume** ton/year	Number of plants	*E. coli* and ACC satisfactory/acceptable	*E. coli* unsatisfactory	ACC unsatisfactory	*E. coli* and ACC unsatisfactory
1	11	3	2	5	1
2	2	0	2	0	0
3	4	1	0	0	3
4	3	1	0	0	2
5	2	0	1	1	0
9	1	1	0	0	0
10	1	0	1	0	0
11	1	0	0	0	1
15	3	1	2	0	0
20	1	1	0	0	0
50	3	0	1	0	2
100	1	0	0	0	1
Total	33	8	9	6	10

*Satisfactory sample = all values observed under m. Acceptable sample = maximum two values are between m and M while the rest of the values are under m. Unsatisfactory sample = any unit exceeds M or if more than two units are between m and M. For *E. coli* m = 50 cfu/g and M = 500 cfu/g. For ACC m = 5 × 10^5^ cfu/g and M = 5 × 10^6^ cfu/g [4], . **Results sorted after the plant production volume 1-100 ton per year

*E. coli* was more often causing unsatisfactory sample assessment than ACC (9 and 16 respectively of 33 samples) (Table 1), but this difference was not statistically significant (*P* = 0.607, McNemar Test). In total, about one fourth of samples, (0.242, 95% C.I: 0.111;0.423) were satisfactory or acceptable. The 165 units, clustered by 33 establishments, had a median for ACC of 5.6 log_10_ colony forming units, cfu/g and *E. coli* 1.8 log_10_ cfu/g. The mixed-effect model (units clustered by establishments [23]), showed according to our expectation that the cluster effect is strong and 90–92% of variation is between establishments (variance *E. coli* 0.32 and 0.035, ACC 1.522 and 0.135; between and within establishments; respectively). There is a possible trend for *E. coli* with highest values in September and lowest in December and an opposite pattern for ACC, but these are not statistically significant (Table 2). We could not detect possible patterns perhaps because of too small number of sampled establishments. Even high between-cluster variation can mask smaller seasonal effects or perhaps there is no genuine seasonal effect.

**Table 2:** Seasonal effects* for *Escherichia coli* and ACC with December as reference

Month	No of units	*E. coli*				ACC			
		Estimate	SE	t-value	Change	Estimate	SE	t-value	Change
(Intercept)	10	1.71	0.40	4.229	Reference	6.73	0.88	7.648	Reference
November	55	0.27	0.44	0.618	+ 16%	− 1.47	0.96	− 1.540	− 77%
October	90	0.54	0.43	1.269	+ 32%	− 1.10	0.93	− 1.191	− 67%
September	10	0.89	0.57	1.556	+ 52%	− 1.86	1.24	− 1.495	− 84%

* The seasonal effect was analysed by fixed effects from Linear Mixed-effects model for units results for *E. coli* and ACC (Aerobic Colony Count) showing December as reference (Intercept). SE = standard error. All seasonal effects were non-significant

Microbiological quality of wild boar carcasses can vary, giving lowest quality at private handling premises when it comes to the mesophilic counts (< 4.0 log_10_ CFU/cm^2^) while *Enterobacteriaceae* can be similarly satisfactory (< 2.0 log_10_ CFU/cm^2^) at registered and private premises [12]. On the other hand, minced meat of wild boar produced by hunters at their private premises, at registered local retail and at approved premises integrated to a game handling establishment can show the best results from hunters private mincing (mean ACC 6.1, 7.3, 7.7 log_10_ CFU/g, mean *E.coli* 2.1, 2.4, 2.1 log_10_ CFU/g, respectively) [24]. Our samples are taken between September and December which can show lower contamination level than summer period could do [25].

Samples are representing establishments over the whole wild boar populations area in Sweden, but the sample size is limited which must be taken in consideration. Moreover, there can be potential sample handling effects caused by the time of thawing at the laboratory. The assessment of STEC in minced meat of wild boar would have been beneficial from consumer safety point of view but not included in this study.

Our results show a high number of non-compliant samples. Nevertheless, we conclude that it is possible to produce minced meat of wild boar of good bacteriological quality. Since wild boar may harbour many bacteriological zoonotic hazards, hunters should be informed of the need of good shooting skills and evisceration routines as well as further handling of the carcass on the field. All actors along the chain must strive for a continuous decreasing chilling curve. Food business operators must focus on control of incoming carcasses and educate staff in hygienic handling at every production stage including proper labelling according to requirements [4]. In case of unsatisfactory results of in-house sampling, effective corrective action should be implemented. The competent authority has the responsibility to verify these activities.

## Data Availability

The datasets used and/or analysed during the current study are available from the corresponding author on reasonable request.
